# Ten unanswered questions in multimodal communication

**DOI:** 10.1007/s00265-013-1565-y

**Published:** 2013-06-21

**Authors:** Sarah R. Partan

**Affiliations:** School of Cognitive Science, Hampshire College, Amherst, MA 01002 USA

**Keywords:** Animal communication, Signal redundancy, Multisensory integration, Noise, Phenotypic plasticity, Climate change, Anthropogenic environmental change

## Abstract

The study of multimodal communication has become an active and vibrant field. This special issue of Behavioral Ecology and Sociobiology brings together new developments in this rapidly expanding area. In this final contribution to the special issue, I look to the future and discuss ten questions in need of further work, touching on issues ranging from theoretical modeling and the evolution of behavior to molecular mechanisms and the development of behavior. In particular, I emphasize that the use of multimodal communication allows animals to switch between sensory channels when one channel becomes too noisy, and suggest that a better understanding of this process may help us both to understand the evolution of multisensory signaling and to predict the success of species facing environmental changes that affect signaling channels, such as urbanization and climate change. An expanded section is included on the effects of climate change on animal communication across sensory channels, urging researchers to pursue this topic due to the rapidity with which the environment is currently transforming.

## Introduction

Multimodal communication is ubiquitous. The ability of organisms to produce and perceive signals in multiple sensory channels profoundly impacts the evolution of signaling behavior, sensory and perceptual physiology, and even speciation and survival. Darwin (1872) anticipated the importance of communication via multiple sensory channels and early ethologists described these multimodal animal signals (e.g., Tinbergen 1959; Marler 1965), yet the topic did not enjoy widespread attention until recently. Part of this new interest has been spurred by theoretical advances: in the 1990s, a series of theoretical papers appeared on the evolution of multiple traits (although they primarily discussed multiple traits within one sensory channel, e.g., Møller and Pomiankowski (1993), Schluter and Price (1993), Iwasa and Pomiankowski (1994), Johnstone (1995, 1996), and Rowe (1999)); attention was drawn specifically to multimodality with papers explicitly discussing multimodal signaling (e.g., Partan and Marler 1999; Rowe and Guilford 1999). In addition, empirical approaches were developed for examining each component of multimodal signals separately, enabling the dissection of the signal to test for interactions among components and to investigate the effects of signaling across several modalities in an integrated fashion (for early work in this area, see Stratton and Uetz (1981), Marples et al. (1994), Terrick et al. (1995), Hughes (1996), Rowe and Guilford (1996), and Scheffer et al. (1996)). Since the 1990s, there has been a substantial increase in the number of papers published on multimodal communication (meta-analysis data on publication rates is in Leonard et al. (2011)). There is now a great deal of interest in studying multimodal communication, particularly in using experimental approaches capable of attributing cause and effect, and in grappling directly with evolutionary consequences of utilizing multiple signal channels. As the papers in this Special Issue on Multimodal Communication demonstrate, the field of multimodal communication has rapidly become a vibrant and productive area of research.

In this essay, I propose ideas for future directions for the field by suggesting ten areas ripe for further work (Table 1). There are certainly more than ten unanswered questions in the field of multimodal communication, but these are a sample of some of the more pressing or intriguing questions. Although the focus of this essay will be primarily on animal signals, plants also use multimodal signals that evolve under many of the same pressures, such as floral visual and olfactory signals used to attract pollinators (Odell et al. 1999; Leonard et al. 2011); the ensuing ideas may be broadly applicable across organisms.

**Table 1:** Ten unanswered questions in multimodal communication

1. Are multimodal signals fundamentally different than unimodal multicomponent signals, requiring their own models?
2. Can we create more comprehensive diagrams or visual models of multimodal communication?
3. Are multimodal signals more likely than unimodal signals to facilitate speciation?
4. What are the costs and constraints of multimodal signaling, and are they similar across taxa?
5. Is there a common mechanism for multisensory integration across channels?
6. How do temporal factors in signal production, transmission, and perception affect multimodal communication?
7. How does multimodal communication develop and is there a common developmental trajectory across taxa?
8. How can genomics and advanced sequencing techniques help us to advance our understanding of multimodal communication?
9. Applications: Can we apply what we learn about multimodal communication in animals to improve their care and handling, or to better the human condition?
10. Integration of basic and applied work: Does the use of multimodal communication allow organisms to survive rapid environmental change more successfully than they would otherwise?

The first eight questions below cover a range of basic science issues, including both theoretical and empirical work. The ninth question explores applied work. The final question integrates basic and applied science to address the question of whether the use of multimodal signaling allows organisms to survive environmental change more successfully than they would otherwise. I expand this final section to discuss and encourage further work on two current case studies: urbanization and climate change. These topics are given emphasis because of the staggering rapidity with which the earth’s environment is currently transforming, and the important role that scientists can play in documenting and potentially helping to ameliorate some of the more detrimental effects of current and upcoming changes.

## Ten questions

1. Are multimodal signals fundamentally different than unimodal multicomponent signals, requiring their own models?

In terms of signal production and perception, multimodal signals (those with components in different sensory channels) differ from unimodal multicomponent signals (those with multiple components all in the same sensory channel) only in that they utilize more than one sensory/perceptual channel. Does this require that we develop new models to understand them? Many of the mathematical models developed to explain the evolution of complex signals focus on multicomponent unimodal signals, such as multiple visual traits used in mate choice in birds (see modeling references in introduction above, reviewed in Bradbury and Vehrencamp (1998), Candolin (2003), Grether et al. (2004), and Otovic and Partan (2009)). We need work devoted to exploring whether these models are applicable to *multimodal* signals and to contexts other than sexual selection.

I would argue that multimodal signals can function in fundamentally different ways than multicomponent unimodal signals can, partly in that employing more than one sensory channel can aid in overcoming noise in one of the channels, if the animal is able to switch between channels (Brumm and Slabbekoorn 2005; Hebets and Papaj 2005; Partan and Marler 2005; discussed further under question 10 below). In another contribution to this special issue, Uy and Safran suggest another unique feature of multimodal signals: since each signal channel transmits at a different rate through the environment, multimodal signals allow sequential assessment in a manner that unimodal ones do not (temporal issues will be discussed below under question 6). In addition, due to the fact that they stimulate two separate sensory systems, multimodal signals might be more readily detected and remembered by receivers than multicomponent unimodal signals, as suggested by Rowe (1999).

To successfully overcome noise by switching from reliance on one channel to reliance on another, the signal components in the two channels must be at least partially redundant in meaning. The evolution of this type of signaling system has been modeled by Ay et al. (2007) in the context of robustness of signal design: a signal is robust if it can withstand partial occlusion, which it can if it contains separate modular clusters that are at least to some extent correlated in meaning. Redundancy between channels is important early in the evolution of the signal, but it is also required in present time if it is to allow an individual to shift, on a facultative basis, between channels in the presence of noise. The importance of redundancy in allowing multimodal signals to overcome noise leads to the prediction that multimodal signals might also be different from unimodal multicomponent signals in the tendency for the different components to carry redundant or nonredundant information. There are many examples of redundant (and nonredundant) multimodal signals (reviewed in Partan (2004a) and Partan and Marler (2005)). Yet in a review of multiple signals used in mate choice, which focused primarily on multicomponent unisensory signals, Candolin (2003) suggested that multiple signals are more likely to be made up of nonredundant than redundant components (“multiple messages” rather than “backup signals”; Johnstone 1996). It would be interesting to conduct comparative surveys to determine whether multimodal signals may in fact be more likely to be redundant, whereas unimodal multicomponent signals more likely to be nonredundant. If so, we need to take this into consideration while creating new models.

In the current special issue, Wilson et al. explore theoretical factors that may promote multiple and multimodal signaling above unimodal signaling. They suggest that in basic models of honest communication, including costly signaling models and “cheap talk” models, multiple signaling confers no benefits beyond single signals. However, when constraints are added to the models, such as the constraint that a unitary signal channel may not have sufficient bandwidth to impart all the information needed by receivers, or the constraint that noise occurs differentially among channels, multiple and multimodal signaling may be favored. Similarly, Ay et al. (2007) showed that pressures for signal complexity and for robustness would favor the evolution of multimodality.

**Fig. 1:**
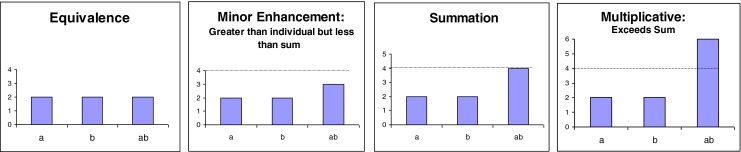
Idealized responses to redundant signal components **a** and **b** separately, and to the multimodal combination **a** and **b**. The multimodal signal can elicit the same response as the separate components (equivalence), or an enhanced response (minor enhancement, summation, or multiplicative enhancement). From Partan (2004a) in The Handbook of Multisensory Processes, edited by GA Calvert, C Spence, and BE Stein, with permission from The MIT Press

There are many kinds of models, requiring different methods of design and testing, varying from game theoretic and other ultimate models (such as those discussed above by Ay et al. (2007) and Wilson et al. (2013)), to proximate physical models (Webb 2001) and visual depictions (visual diagrammatic models will be discussed in the next section, question 2). In the development of mathematical models for proximate questions about multimodality, it would be helpful to design models that can predict how multimodal components could be combined or that can predict response outcomes of different combinations (Partan and Marler 2005). Sih et al. (2011) discuss how signal detection theory can be used to help us to make predictions for how different cues or cue components affect discriminability in different environmental conditions. It would also be helpful to determine quantitative thresholds for signal response categories, that would allow one to distinguish among, for example, three types of multimodal enhancement for redundant signals (Partan 2004a): minor enhancement (less than the sum), summation (or “additive”), and multiplicative enhancement (Fig. 1). Castellano (2010) modeled additive and multiplicative signal interactions in a model of mate choice based on sequential assessment of cues, showing that signals used in species recognition can amplify the effects of mate choice on sexual selection. To help us develop new models explicitly for multimodality, improved visual communication in the form of diagrams may be useful, discussed next.

2. Can we create more comprehensive diagrams or visual models of multimodal communication?

Images are powerful: they can open the mind to new ideas, yet they can also constrain thought. Diagrams and other visual models can be used as tools for learning, communicating ideas, solving problems, improving understanding, and, indeed, shaping how we think about phenomena—such as the communication process.

**Fig. 2:**
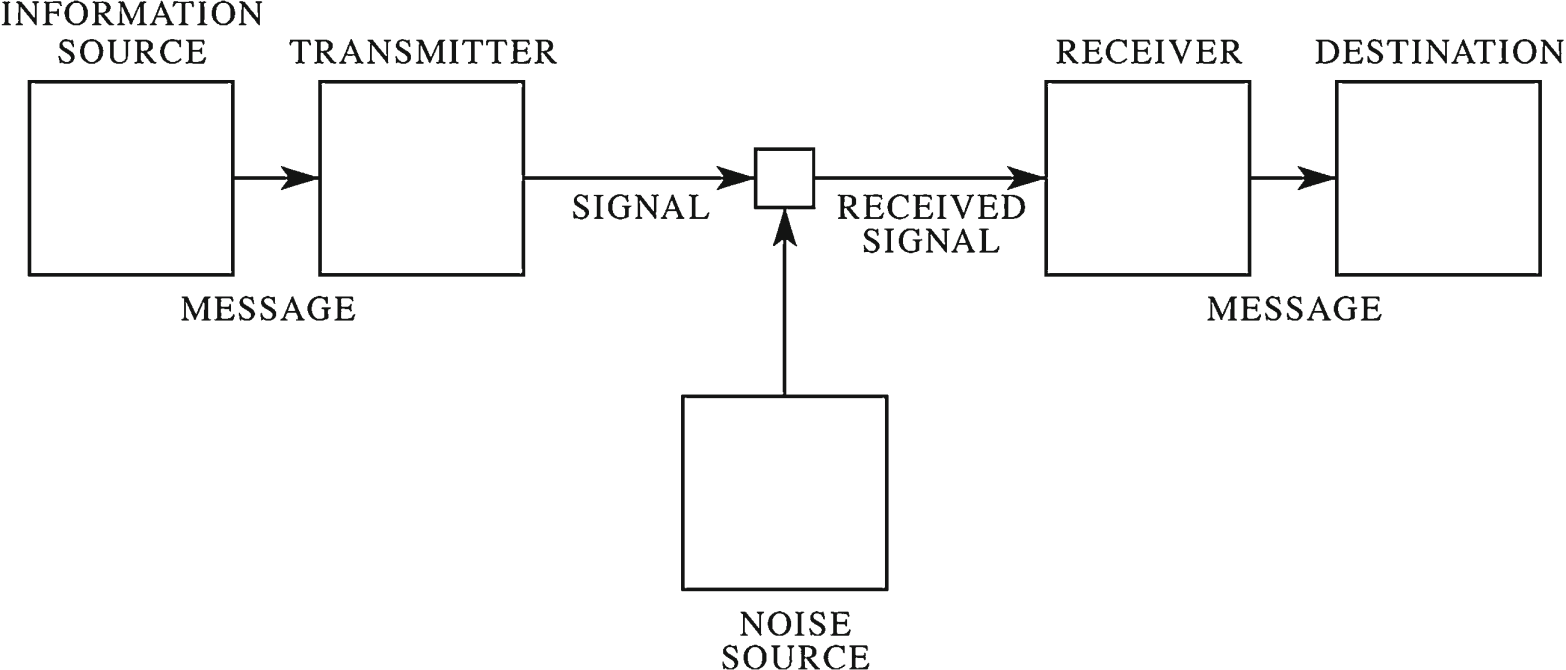
Basic model of communication. From Shannon (1948) in the Bell Systems Technical Journal with permission from Alcatel-Lucent

To develop models for multimodal communication that adequately represent differences between multimodal and unimodal signaling, it would help to have more explicit diagrams of multimodal communication that incorporate multiple channels. Traditionally, communication has been diagrammed as occurring between two actors, a sender and a receiver, through one channel. In 1948, Claude Shannon famously produced a diagram (Fig. 2) that depicts six boxes: an information source (later referred to by others as “sender” or “actor”), a transmitter (which produces the signal), a channel, a noise source that impacts the channel, a receiver (akin to sensory receptors), and a destination (later referred to as the “receiver” or “recipient”).

Although it is understood that models and diagrams simplify reality (in fact this simplification is one of the great benefits of models), it is easy to forget about the complexities of communication when studying a simple diagram. Real communication, in contrast to the diagram in Fig. 2, actually involves multiple signal components and multiple sensory channels and, usually, more than two interactants. In the text of his paper, Shannon (1948) in fact wrote that the information source is not necessarily unitary but may include several variables and also combinations of variables, such as light, color, and sound in a television. The power of a diagram, however, is to replace the complexity found in the text with the simplicity of an image, and when we view the diagram we tend to forget that the “information source” was intended to be multidimensional.

During real communication in situ, there are many factors at play. In addition to the multifaceted signal itself, the context of the signal also has a profound effect on the interaction, including the number of individuals involved and their history, as well as physical features of the surrounding environment (Partan and Marler 2002). Even when dealing with only two interactants, it can be difficult to determine which one is the “sender” and which the “receiver,” since both often produce behavior at the same time, in an active, dynamic, and collaborative interchange. Shanker and King (2002) suggested that the framework of information transfer limits our imagination and our understanding of the dynamic interactions that occur during communication. As an antidote, they refer to communication as a “dance” and employ Dynamic Systems Theory to analyze it. Although there are many good counterarguments that an informational approach can indeed describe even complex interactions (e.g., Zentall 2002), it may still be fruitful to challenge ourselves to think in new and creative ways about how to describe communication.

It is difficult to create new diagrams of communication that elegantly and adequately express the full communication process, incorporating multiple facets of the signal, multiple channels, multiple interactants, dynamic factors, and effects of the environment, without becoming so complex as to lose visual effectiveness. For those interested in taking up the challenge to do so, it may be useful to consult an interesting model from the field of journalism by Westley and MacLean (1957), or diagrams from transactional models of communication (e.g., Barnlund 1970), or reinterpretations of the functional cycle diagram of von Uexkull (1934) that depict a dynamic view of communication (Burghardt 1998; Partan and Marler 2002). It would also be interesting to consider visual representations from a quantitative perspective, such as Smith and Evans suggest in their contribution to this special issue, by graphing 3D receiver response plots given different degrees of variability of a multimodal signal across channels. Improved diagrams could help communicate ideas across fields, such as empirical scientists and theoreticians collaborating to develop mathematical models of communication, as well as help us to design new studies and better methods for data collection from multiple parties and multiple channels.

3. Are multimodal signals more likely than unimodal signals to facilitate speciation?

Environmental differences that affect signal transmission can promote speciation (Endler 1992; Boughman 2002), to be discussed further in question 10 below. Schluter and Price (1993) suggested that differences between environments in how traits are detected should determine which trait is relied upon more, leading to divergent selection for multiple traits between different environments. Likewise, Candolin (2003) suggested that multiple cues can lead to greater levels of speciation if subgroups of individuals come to rely differentially on different cues, leading to assortative mating. Evidence for these ideas in terms of multimodal signals comes from Seddon et al. (2008), who found that the degree of signal complexity in antbirds, including coloration and song, was positively associated with the number of species per genus, and from Uy et al. (2009), who examined how visual and auditory signals of flycatchers are used in species recognition, suggesting that multimodality may promote rapid speciation by enabling effective communication under changing conditions. This area is ripe for further investigation, as was suggested by Bro-Jørgensen (2010).

4. What are the costs and constraints of multimodal signaling, and are they similar across taxa?

We need more empirical research on the costs of multimodal signaling (Munoz and Blumstein 2012), which can potentially include increased energetic expenditure and increased predation risk (Partan and Marler 2005). We know neither the relative energetic expenses of signaling in the individual sensory channels, nor the combined expense of multimodal signaling (Partan and Marler 2005). In sexual selection models, energetic costs can be considered indirect costs, in that they may or may not ultimately affect the lifetime reproductive success of an individual (Kotiaho 2001). To assess direct costs, one must collect data on how a trait is connected to a direct fitness consequence such as predation or parasitism (Kotiaho 2001). Roberts et al. (2007) report one of the few experimental tests of this in a multimodal system: they measured predatory response rates of jumping spiders to playback of signals from their prey, wolf spiders, and found that multimodal signals elicited faster predatory responses than did unimodal signals. This direct cost of multimodality, that of increased conspicuousness to predators, should be tested across taxa and across different modality combinations.

Constraints of multimodal signaling are underexplored, particularly in the sense of anatomical and physiological constraints that one channel puts on another channel. For example, vocalization, breathing, and posture are intimately connected, causing changes in visual appearance during acoustic signaling behavior. Conversely, changes to posture affect the quality of sound produced. At an anatomical level, body size is related to pitch: larger individuals can make lower frequency sounds (Morton 1977), and, presumably, larger visual gestures. In birds, for example, beak size and gape size of beaks during singing has been shown to affect the rate and frequency of the song on a moment-to-moment basis (e.g., Westneat et al. 1993; Podos et al. 1995, 2004; Derryberry et al. 2012). Co-development, and co-evolution, of body size, vocal tract, and posture (both as a visual cue and as a contributor to vocal quality) would be quite interesting to explore across taxa.

5. Is there a common mechanism for multisensory integration across channels?

This question covers a suite of related issues, because the word “mechanism” can be used at the physiological level or at the behavioral level, and it can also be used for signal perception or production. At the physiological, perceptual level, the study of neural mechanisms for the perceptual integration of multisensory information is a highly active area of research; multiple important locations and mechanisms have been discovered for the neural integration of information from the different sensory systems. For reviews and research in this area along with ideas for further work, see the contributions published in the Handbooks of Multisensory Processing edited by Calvert et al. (2004) and by Stein (2012).

At the overt behavioral level of signal production, signals from multiple sensory channels can be integrated or combined in multiple ways (e.g., they can be redundant or nonredundant, with many other subtleties; Partan and Marler 1999). An open question is whether signal components are combined across sensory channels in similar ways irrespective of sensory modality. For example, is it equally likely that multimodal signals will be redundant or nonredundant regardless of which sensory channels are involved, or are particular pairings of channels more effective in redundant or in nonredundant combinations? Otovic and Partan (2009) provide some data on this question (see their table 6). A related issue is whether there are predictable combinations of sensory modalities that work best in particular contexts or environments, depending on external factors such as transmission properties of that environment. The “active space” of a signal (how far it travels or can be perceived) differs by sensory channel: auditory and, depending on the environment, visual signals can reach distant receivers, and are often used for “public” or “broadcast” displays, whereas tactile and, depending on volatility and wind conditions, olfactory signals generally require closer receiver distances and can be used for more private communications. Wilson et al. (2013) discuss this distinction and suggest that, in the context of mate choice, public channels would be used for displaying globally preferred qualities, whereas private channels would be used for signaling features that are idiosyncratically favored by a particular receiver. Finally, sometimes a signal component in one channel is used to attract attention, and a component in another channel carries the message (e.g., audio signals alerting a receiver to visual signals; Grafe and Wanger 2007). Is any sensory modality equally as good as any other for either of these roles, depending on the context, or are there predictable combinations of modalities that work best for each role? Some of these questions have been discussed previously (e.g., in Partan and Marler 2005) but all need empirical work.

6. How do temporal factors in signal production, transmission, and perception affect multimodal communication?

Multimodal signal components can be produced synchronously or asynchronously, and due to varying signal transmission properties of the different channels through the environment, even synchronously produced components can be received sequentially (Wickler 1978; Uy and Safran 2013; reviewed in Partan and Marler (2005)). This is particularly the case for chemical signals, to be discussed further below, because they transmit quite differently from the other channels. Furthermore, due to differential speeds of neural processing of stimuli from different senses, even signal components that arrive simultaneously can be processed sequentially (Bremner et al. 2012a). In some cases, differences in the speed of internal signal transduction can make up for differences in environmental signal transmission: sound is transduced more rapidly than light, somewhat making up for the fact that sound travels much more slowly through air than does light (Spence and Squire 2003). We need to do more work to understand how these timing issues affect multimodal communication, both at the overt behavioral level and at the level of neural integration.

At what point of temporal separation should an asynchronous multimodal signal be considered two separate unimodal signals? One approach to answering this question is to examine signal structure. Elias et al. (2006), for example, gave a detailed report of the sound-producing mechanisms in wolf spiders, showing that multicomponent seismic signals can be either simultaneously or sequentially produced, and advocating for further work describing signal production mechanisms. Another approach to answering this question is to test receiver perception. Narins et al. (2005) tested whether receivers required exact temporal synchrony between vocal (advertisement call) and visual (vocal sac expansion) components of male frog displays. Although exact synchrony was not required to elicit aggressive responses from resident males, some degree of overlap between the two signal components was necessary; signals with temporal delays that did not include an overlap were much less effective. This suggests that successful binding of multimodal inputs requires some degree of temporal coordination. It leaves open the question of whether sequential multimodal stimuli are processed differently than simultaneous ones. Uy and Safran (2013) suggest that many simultaneous multimodal signals are in fact assessed sequentially, and that this may be a benefit rather than a disadvantage. They conducted a field playback study to test conspecific recognition in two different sets of flycatcher subspecies, finding that for one sister pair of taxa, song was apparently assessed before color, whereas for the second pair of taxa, color was assessed early along with song. Uy and Safran suggest that differences in the timing of signal assessment may be related to the extent of divergence between song types of the two taxa, or may be related to differences in environmental transmission properties, in that the first environment occluded visual transmission more than the second.

Olfactory signals provide a particularly interesting and challenging problem for temporal integration of multimodal signaling. The active space of a chemical signal differs considerably from the other sensory channels: chemicals travel more slowly, last longer, and vary greatly in spatial transmission depending on environmental conditions. In addition, they are tough to study and have been underrepresented in the communication literature (Coleman 2009). However, chemical signaling is universal across taxa (Wyatt 2010) and olfactory signals are often produced in tandem with signal components in the other speedier channels (Partan and Marler 2005). How are olfactory cues integrated during communication interactions, behaviorally, and how are olfactory components perceptually integrated with the other channels in the brain? This is a particularly important question in the context of development, since infants are highly influenced by olfactory stimuli, and indeed in mammals olfactory chemosensors become functional earlier in development than do other sensory systems (Schaal and Durand 2012). Work is increasing on olfaction and multisensory processing, with further interest predicted (Walla 2008). For example, a renewed interest is developing on the use of olfactory cues in concert with visual, tactile, and auditory cues during multimodal sexual behavior in primates (see earlier work by Beilert (1982) and Goldfoot (1982), and recent work by Jones and Van Cantfort (2007), Clarke et al. (2009), Higham et al. (2009); see also Semple and Higham (2013), for discussion of work currently in press on olfaction in primates). The role of chemical cues in multimodal communication has also been studied in crustaceans (reviewed by Hebets and Rundus (2011)). Given the importance of chemical signals in mate choice, predation, and other ecologically salient interactions, we need more work on how they are used in multimodal communication, along with a better understanding more generally of temporal issues in multimodal signaling.

7. How does multimodal communication develop and is there a common developmental trajectory across taxa?

The development of multimodal signal production and perception is understudied, but is of crucial importance in understanding proximate mechanisms as well as ultimate functions of behavior. There is a body of work on the development of audiovisual speech perception in humans (reviewed in Soto-Faraco et al. (2012)) and related work on the development of cross-modal perception in nonhuman primate communication (reviewed by Ghazanfar (2012, 2013)), but outside of these areas there has not been a lot of work on the ontogeny of multimodal communication. In the animal communication literature, developmental work has tended to focus on the acoustic channel on its own: song learning in songbirds is the classic example, and vocal perception in frogs is also garnering interest (Baugh et al. 2012). Future work could incorporate a focus on other signal channels in addition to the acoustic one.

In contrast to the paucity of work on development of multimodal *communication* specifically, there is a wealth of work on the development of multimodal *perception* more broadly. Research on multisensory perceptual development has recently been gathered together in a volume edited by Bremner et al. (2012b). At a basic level, different sensory systems develop at different rates (Turkewitz and Kenny 1982) and multisensory information is of crucial importance to developing organisms, both in humans (Walker-Andrews 1997; Lickliter and Bahrick 2004; Flom and Bahrick 2007) and in other animals (e.g., nonhuman primates (Zangenehpour et al. 2009), birds (Honeycutt and Lickliter 2001; Lickliter et al. 2002)).

One long-standing question regarding perception is whether infants are born perceiving the world through unisensory systems and subsequently develop the ability to integrate across sensory channels, as initially proposed by Piaget, or whether they are born perceiving information from all senses blended together and later develop the ability to differentiate among them, as proposed by Eleanor Gibson and supported with recent evidence (discussed in Bremner et al. (2012a)). It may be that both views are partially correct, in that redundant information is perceived multimodally from birth (Bahrick and Lickliter 2000), likely based on specific prenatal sensory experience (Lickliter 2011), but correspondences among nonredundant multimodal stimuli have to be learned (Bremner et al. 2012a).

Much work remains to be done to understand the role of factors such as environment and experience (see Lewkowicz and Ghazanfar (2009)), familiarity, attention, and the integration of sensory channels in multimodal development, across taxa.

8. How can genomics and advanced sequencing techniques help us to advance our understanding of multimodal communication?

The rapid development of new DNA sequencing techniques is allowing the field of genomics, along with evolutionary and behavioral genetics, to expand at a fast pace. Contemporary, real-time evolution can now be studied more directly than ever before (Losos et al. 2013), and massive databases are being compiled that include data on morphology, behavior, and genomes (Losos et al. 2013). There are two main approaches to DNA sequencing: one is to sequence the entire genome, which provides a static look at the entire DNA sequence of an organism, and the other is to sequence the transcriptome, the portion of the genome that is currently being transcribed into RNA. In contrast to the whole genome, the transcriptome is variable, reflecting effects of environment, because it only contains those genes that are currently being expressed. Both approaches to sequencing can be used to explore the genetic basis of behavior.

Next-generation sequencing (NGS) is a new method of sequencing that is competing with its predecessor, microarray analysis (Hurd and Nelson 2009). Microarrays have provided stellar opportunities to learn about the genetic basis of behavior, such as the work by Cummings et al. (2008) who used microarrays to examine gene expression in female swordtail fish during mate choice behavior, and found that rapid changes to gene activity occur as females assess social options. Microarrays have limitations, however, such as being expensive and requiring a priori knowledge of the genome (Hurd and Nelson 2009). The new NGS methods are less expensive and do not require a priori knowledge, so can be used to ask unbiased (“hypothesis-free”) questions and can be conducted with non-model organisms (i.e., those whose genome has not been sequenced). NGS uses rapid parallel processing (“ultra high throughput”) of DNA, producing massive amounts of sequence data quickly (Ellegren 2008). This method can be used also on RNA, called “RNA-Seq” (Hitzemann et al. 2013), to sequence the transcriptome.

With the development of NGS, we have a way to rapidly examine the genome as well as current gene expression across thousands of gene sequences, allowing comparison of multiple organisms across a variety of behavioral contexts. Research applying these techniques to complex behaviors and behavioral phenotypes is quite new, but promising results are reviewed in Wong and Hofmann (2010), who predict that due to the relatively low cost and high potential of the newer techniques they will increase in usage. An elegant demonstration of the power of next-generation sequencing methods to help us understand the genetic underpinnings of behavior was recently published by Weber et al. (2013), who found that the genetic basis of natural burrow-digging behavior in mice can be localized in the genome, and furthermore that the behavior is made up of discrete modules, each having a separate identifiable genetic locus.

In terms of animal communication specifically, a great deal of genetics work has been done on the song control system of the zebra finch, a new model organism whose genome has recently been sequenced (Warren et al. 2010). The genome sequences along with the new methods are enabling great strides in the discovery of the genetic basis of communication in songbirds (e.g., Lovell et al. 2008; Clayton et al. 2009; Dong et al. 2009; Gunaratne et al. 2011). Although the overwhelming majority of the work on the genetics of the zebra finch song system has focused purely on the auditory domain, there are a few single-gene expression studies that have addressed multimodal questions (Kruse et al. 2004; Avey et al. 2005), paving the way for future genomics work on multimodal communication.

The potential of gene expression studies and advanced sequencing methods to uncover mechanisms that underlay both the production and the perception of multimodal signals is exciting. It is also worth remembering that there are many challenges involved in seeking the genetic basis of complex behavior; behavior is not just affected by genes but is also highly dependent on the environment, for example, including both the immediate and the prior developmental environment (Hoekstra 2010). Mechanistic work, particularly with gene expression, requires extremely controlled external and internal environments. The choice of behavior to study is key: one needs a behavior that is reliably measured and has a heritable component; it is important to conduct a breeding experiment to assess the heritability of the behavior (Hoekstra 2010). In addition, to measure gene expression during behavior, one has to be very lucky (or persistent) to sample just the right tissue at just the right moment, given the rapid time course of gene expression. There is much challenging and exciting work to be done in this area.

9. Applications: Can we apply what we learn about multimodal communication in animals to improve their care and handling, or to better the human condition?

The field of multimodal communication, like many topics in animal behavior, has focused primarily on basic rather than applied science. An understanding of how animals use multiple sensory systems and channels in communication could help, however, with issues such as animal training, management, and welfare. For example, understanding how animals integrate information from multiple sources can help address the question of whether dogs and other domesticated animals should be trained using visual cues, auditory cues, or both (see Braem and Mills 2010). Knowledge of what combination of sensory channels is used by particular taxa may also help us to create captive animal facilities that are quiet enough, acoustically, visually, and/or chemically, to allow for successful communication (Grandin 1995; Coppola et al. 2006), or to ensure that animals that are translocated or rehabilitated for release are released in appropriate habitats and prepared to recognize ecologically relevant multisensory stimuli (Munoz and Blumstein 2012).

We also can use our work on multimodal communication to help with human needs. For example, children with developmental impairments or adults rehabilitating after stroke often need intensive language and communication tutoring. An understanding of the relationship between auditory and visual components of communication can help practitioners to develop effective therapies and educational programs (see Massaro (1998) and Massaro and Light (2004); see also Sathian (2012) for references on multisensory rehabilitation for other neurological sensory disorders). As another example, collaboration with the fields of multimodal perception and psychophysics can improve our understanding of how workers such as airplane pilots and air traffic controllers can quickly and effectively integrate multiple sources of information. In another application, Sih et al. (2011) discuss crop pests and why some herbivores are attracted to particular crops but others are not; they suggest that an understanding of the multiple chemical, visual, and tactile cues from crop species, and of the sensory systems and preferences of herbivores, will help us devise strategies to minimize crop damage. Many important applications of multimodal communication research need work.

10. Integration of basic and applied work: does the use of multimodal communication allow organisms to survive rapid environmental change more successfully than they would otherwise?

Environmental change spurs evolution. Rapid environmental change, however, can lead to species demise if the organism is not able to adjust or adapt quickly enough to survive. Sih et al. (2011) provide an extensive synthesis of how organisms are prepared (or unprepared) by their evolutionary history to cope with human induced rapid environmental change, and discuss the relative roles of behavioral plasticity and evolutionary history in coping. Much work has focused recently on the effects of rapid environmental change on morphology and life history traits. Gienapp et al. (2008) review the evidence of trait changes associated with climate change, concluding that most of these effects are likely due to phenotypic plasticity rather than microevolutionary genetic adaptation; Charmantier et al. (2008) and Garant et al. (2008) provide data for this finding in birds. It may be that genetic evolution is constrained in variable environments because of a trade-off between the strength of selection and degree of heritable variability: evolution would be limited by either low selection or low variability (Wilson et al. 2006). It has also been suggested, however, that unpredictable environments should preserve variability in traits (Robinson et al. 2008). This is because there is not one optimal trait or strategy when the environment is unpredictable. Male sheep, for example, should put energy into growing big horns when environmental conditions are good, but in lean times energy should be saved for survival, so variation in strategy (in the tradeoff between investing in sexually selected traits versus survival) would be selected for in fluctuating environments (Robinson et al. 2008). This extensive body of work on how environmental change affects morphology and life history paves the way for behavioral scientists to apply these approaches to communication and other behaviors.

In this special expanded section, I will address the question of whether the use of multimodal communication, in particular, helps organisms to survive rapid change (see Bro-Jørgensen (2010)). I argue that the use of multiple sensory channels is beneficial because it increases flexibility, allowing strategies such as shifting between channels to overcome noise in one of the channels, or using different channels in new and changed environments that have different transmission properties. Below I will first discuss multimodal shifts between channels; I will then move to two case studies of rapid environmental change: urbanization and climate change.

### Multimodal shifts

Increases in environmental noise (in any sensory channel) may impair signal transmission; organisms can respond to this by adjusting various features of their communication signals to overcome the noise. As stated above, multimodal signaling allows individuals to shift between sensory channels when they encounter noise in one of the channels (Brumm and Slabbekoorn 2005; Hebets and Papaj 2005; Partan and Marler 2005; van der Sluijs et al. 2011), termed a “multimodal shift” by Partan et al. (2010). A multimodal shift involves moving from reliance on one channel (or configuration of channels) in one context to relying on a different channel (or configuration) in another context. As noise permeates one sensory channel, the ability to shift among channels allows an individual—either a sender or receiver—to continue to convey or perceive the message as long as the channels transmit at least partially redundant information. This can be thought of in terms of the reliability or uncertainty of information in each channel: as the uncertainty in one channel increases, the other channel becomes more important (see Massaro (1998), Munoz and Blumstein (2012)). Smith and Evans (2013) describe a heuristic graph for visualizing signal performance in multiple channels in the face of various degrees of noise in each channel, which may be useful for understanding and predicting signal modifications in noise.

A multimodal shift is a type of phenotypic plasticity, and is similar to the phenomenon of “sensory plasticity” in which one sensory channel is boosted when another is stunted (Chapman et al. 2010). However, sensory plasticity usually is described in terms of development, such that an organism deprived of sensory input in one channel during an early critical period will develop more elaborate neural processing for another channel (Berardi et al. 2000), providing it with better perception in the latter channel for the remainder of its life. Fish raised in low light, for example, rely more on chemical than visual cues for foraging later in life (Chapman et al. 2010). This sensory plasticity differs from the multimodal shifts discussed below in that the latter are short-term changes, occurring when an organism encounters a changed environment, rather than life-long changes.

Short-term multimodal shifts have been documented in captive animals: chimpanzees modify signal modality depending on the attentional focus of their caretaker, using more visual signals when caretakers face them and more audio signals when caretakers face away (Leavens et al. 2010); chickens modify signal modality depending on the attentional focus of the alpha male, using more silent than vocal displays when the alpha is attentive (Smith et al. 2011); spiders reduce visual signaling behavior in the dark (Taylor et al. 2005; Wilgers and Hebets 2011); and stickleback fish rely more on visual than olfactory cues for mate choice in clear water, but in turbid water the olfactory cues increase in importance (Heuschele et al. 2009).

Multimodal shifts have only rarely been documented in the field, however. It has been suggested that frogs living by noisy streams may rely more on visual signals than do those in quiet areas (Hödl and Amézquita 2001), and that when jumping spiders court in open areas they use visual signals whereas when they court in the dark of the nest they use vibrational signals (Jackson 1992). Robins have been found to sing at night, rather than during the day, in areas with high daytime noise (Fuller et al. 2007). It would be quite interesting to study this system further to investigate whether those robins singing in the dark may modify or omit any of the visual accompaniments of their behavior, showing a multimodal shift in signal production between light and dark conditions. In experimental playback work, gray squirrels living in relatively urban areas responded more to visual tail flagging components of simulated alarm signals than did rural squirrels, whereas squirrels in the two habitats responded equally to audio components of the signals (Partan et al. 2010), suggesting that a multimodal shift in emphasis may occur between habitats in squirrels.

More work needs to be done to study multimodal shifts in the field, to discover whether species or individuals that make extensive use of multimodal communication can survive environmental change better than those who do not, due to this potential to shift between channels when one channel is too noisy. The degree of benefit gained from the ability to make this shift may depend on whether the signal components in the two different channels are redundant or nonredundant: as discussed in question 1 above, if the components are redundant, then the animal benefits by being able to transmit the same message when switching to the other channel. However, if they are nonredundant, the unique information in the noisy channel will be lost, impairing the ability of the signal to transmit the whole message. One very interesting implication of this is that redundant multimodal signals may be more resilient in the face of environmental change than nonredundant multimodal signals; this should be tested.

### Case study: urbanization

The current increase in global urbanization provides a compelling case study for examining how organisms adapt to environmental change. Urbanization affects all aspects of animal behavior, including communication (Rabin and Greene 2002; Tuomainen and Candolin 2011; Ryan and Partan 2013). As the world undergoes unprecedented transformation of natural areas to urban ones, the ability of animals to adjust their signaling behavior to crowded, noisy, and polluted environments will be key for their future survival.

Because sensory perception systems can drive the evolution of signals (due to sensory drive; Endler 1992), when environments change in ways that affect signal transmission, and therefore sensory perception, communication systems can begin to diverge, potentially even leading to speciation (see Boughman (2002) review). Urbanization, through the introduction of acoustic, visual, and chemical pollution, has high potential to affect signal transmission. The role of the environment in signal evolution is well studied with regard to light, natural habitat, and visual signals, particularly in fish and lizards (Endler 1992; Fleishman 1992; Ord et al. 2007, 2011), and with regard to attenuation of sound, particularly in birds, frogs, and insects (Morton 1975; Wiley and Richards 1978; Ryan et al. 1990; Forrest 1994). However, less is known about the influence of other aspects of the environment (such as novel urban challenges) on signal evolution, and the role of the environment in the evolution of multimodal communication in particular is not well studied (Bro-Jørgensen 2010; but see Seddon et al. (2008) and Uy et al. (2009), discussed in question 3 above). To understand the evolution of multimodal communication, we should learn how each component of a signal is affected by environmental pressures (Brumm and Slabbekoorn 2005).

Environmental changes such as increased noise can lead animals to alter the timing or structure of their signals. Noise has been shown to cause shifts in signal emphasis within a single sensory channel: in environments with high anthropogenic acoustic noise, animals may adjust the pitch (Rabin et al. 2003; Slabbekoorn and Peet 2003), amplitude (Brumm 2004; Warren et al. 2006), duration (Brumm et al. 2004; Foote et al. 2004), or rate of vocalizations (Sun and Narins 2005; Partan et al. 2010); and in “visual noise” (e.g., wind-blown vegetation) or low light, they may adjust the speed, intensity, or duration of visual displays (Fleishman 1992; Peters et al. 2007; Ord et al. 2007, 2010). Importantly, even though some animals can shift their signaling behavior to attempt to overcome urban noise, they may still experience a drop in reproductive success in urban environments despite these modifications (Halfwerk et al. 2011a, b).

Very little empirical fieldwork on multimodal communication has been done in the urban environment (but see Partan et al. (2010) for a comparison of the response of urban and rural gray squirrels to multimodal playbacks, discussed above); given the rapid increase in urban expansion, this should be a priority.

### Case study: climate change

Climate change provides a second case study of rapid environmental change and how it can influence behavior. If climate change differentially affects signaling channels, then it has the potential to profoundly influence how organisms use multimodal communication. Here I develop an expanded section on the effects of climate change on communication behavior. I argue that climate change can, in fact, differentially affect signaling channels, and urge researchers to study this problem.

Research on the effects of climate change on animals and other organisms has so far generally focused on mortality rates and habitat shifts due to temperature gradient shifts and extreme weather (e.g., Schwartz et al. (2006) in terrestrial environments and Sorte et al. (2011) in the marine environment). Other effects of climate change on behavior include adjustments in the timing of weather-dependent activities such as breeding and migration (reviewed in Wuethrich (2000) and Parmesan (2006)), and even changes to social structure based on changes in resource distribution (Rubenstein 1992).

Few scientists have yet explored the effect of climate change on communication behavior specifically. Here I will discuss how warmer global temperatures and other climate-related changes are likely to affect signal channels. I will begin with the aquatic environment and then discuss the terrestrial environment, since the effects of climate depend in part on the medium for signal transfer (water versus air). Generally speaking, the work in this area deals with one signaling channel at a time, so I will primarily discuss unimodal communication (within a sensory channel). However, as mentioned above, if climate change differentially affects each signal channel, there will be consequences for multimodal communication. Data on unimodal channels is, therefore, germane to the argument that climate affects multimodal communication, and in any case future multimodal work in this area should be based on an understanding of the existing unimodal work.

### Effects of climate change on communication in aquatic environments

Increasing temperatures and other effects of climate change have multiple cascading effects on water quality and clarity, affecting several channels of underwater communication among aquatic animals (van der Sluijs et al. 2011). Endler (1995) outlined a series of potential interactions among changes in temperature, light, and stream size, with multiple factors and multiple outcomes, including alterations to morphology, behavior, and sexual selection.

#### Turbidity and vision

Water turbidity is generally increased by global warming, with consequent decreases in underwater visual signal transmission. There are several reasons that higher temperatures and other climatic changes increase water turbidity. First, warmer water holds less dissolved oxygen than colder water (Stramma et al. 2008). Dissolved oxygen is required for aquatic organisms to live, so the decrease in oxygen can lead to increased death of aquatic life, clouding the water. Second, climate-related increases in rainfall, storms, and snowmelt lead to erosion and runoff into waterways, increasing sediment and therefore turbidity of the water. Third, the increased runoff brings large amounts of agricultural fertilizers to the waterways, spurring eutrophication (increased plant growth, such as algal blooms, stimulated by excess nutrients) and thus further clouding the water and depleting its oxygen content. Higher turbidity itself then leads to a further increase in water temperature because suspended particles absorb heat, intensifying the cycle.

Direct effects of turbidity on communication include decreased visibility, impairing visual communication. Seehausen et al. (1997) showed that cichlid fish in turbid water are less colorful than those in clear water, suggesting that sexual selection is profoundly affected by eutrophication. Experimental simulations of the effects of eutrophication on fish behavior have shown that fish also alter their courtship behavior, in addition to coloration, in turbid water (Wong et al. 2007), with males spending more time on courtship and females more time on mate choice than they do in clear water (Candolin et al. 2007). Gene expression studies of the visual systems of fish have been conducted by, e.g., Fuller et al. (2005), who found that fish can be quite plastic in terms of adjusting the expression of opsins (which determine spectral sensitivity of cones in the eye), depending on the transmission properties of the water in which they were raised. The ultimate consequences of these behavioral and morphological changes are currently unknown, but projections are outlined by Candolin (2009), and eutrophication has been linked to species collapse at a contemporary time scale (Vonlanthen et al. 2012).

Although eutrophication primarily affects shallow waters, fish in open ocean waters may be affected by other climate-related visibility issues. For example, as ocean waters warm, fish may swim at lower depths to seek cooler water. There is less light at lower depths, decreasing color perception and potentially impacting visual communication. We need more research on the effects of temperature, turbidity, and sedimentation on visual communication in aquatic animals, particularly in the field.

#### Underwater acoustics

Climate change also affects the underwater acoustic environment. As the ocean absorbs more and more CO_2_, its acidity increases. In acidic water, lower sound frequencies experience less absorption (Hester et al. 2008), so more distant low-frequency sounds can be heard. This is problematic because industrial noises, such as those from shipping or exploration of the ocean floor, tend to be low frequency, and they can now be heard even more widely across the ocean, increasing ambient underwater noise substantially. Many whales communicate by the use of long-distance, low-frequency vocalizations in ranges overlapping anthropogenic noise (Tyack 2008), and the increase in ambient noise can impair their ability to communicate and find mates. Several studies have documented changes in whale vocal behavior during industrial underwater noise (Miller et al. 2000; Di Iorio and Clark 2010), and one study measured a decrease in stress hormones of baleen whales after a dramatic reduction in shipping traffic noise (Rolland et al. 2012). There are many other effects of noise on fish, reviewed in Popper and Hastings (2009) and Slabbekoorn et al. (2010).

#### Other underwater sensory channels

Chemical signals are very important to aquatic organisms; we need more research on how temperature and turbidity affect underwater pheromonal communication (see Heuschele et al. (2009), discussed above, for work on how turbidity affects the reliance on chemical versus visual cues). In addition, the increased agricultural runoff mentioned above can impair natural chemical communication and mate choice in fish (Fisher et al. 2006). Other effects of climate change on underwater communication may include changes to signal production: in warmer temperatures, electric fish increase their rates of electric organ discharge (Silva et al. 2007).

### Effects of climate change on communication in terrestrial environments

Very few studies have yet directly measured the effects of climate change on terrestrial animal communication. There are several lines of evidence, however, that at least acoustic signal transmission is affected by temperature, wind, and humidity.

#### Airborne acoustics

Garstang et al. (2005) documented African elephant vocal behavior that appeared to be carefully orchestrated to match the moment-to-moment details of the current near-surface atmospheric conditions, including temperature and wind speed, presumably so as to maximize signal transmission distance across the savannah. Generally speaking, elephants tended to call at dawn and dusk, a time when air and substrate temperatures match, but they also regulated calling throughout the day to vocalize specifically when wind speeds were low (Garstang et al. 2005). With climate change, not only do ambient temperatures show an average increase, affecting the match between air and substrate temperatures, but they increase heterogeneously across space, and the increased differentials between areas of cooler and warmer air cause an increase in wind, potentially impairing signal transmission further. Many other animals have likewise taken advantage of the good sound propagation environment at dawn, with dawn choruses (Henwood and Fabrick 1979); we may need new models to predict how they will fare with changing atmospheric and climactic variables.

That wind can impair acoustic signal transmission was shown by playback of pure tones that simulated pika calls. The playbacks were broadcast at the same frequencies and in the same habitats as the pika calls (Hayes and Huntly 2005), and it was found that increasing wind speed increased sound attenuation, with degree of attenuation dependent on wind direction relative to the direction of signal emission. Wind also impairs plant-borne vibrational signals of insects: male treehoppers were found to signal less in windy than in calm conditions and females were less likely to respond during wind than during calm conditions (McNett et al. 2010).

Climate change also affects the moisture content of air, which can affect the transmission of acoustic information. Both temperature and humidity affect sound absorption (Wiley and Richards 1978). In habitats with higher sound absorption, birds have been found to lower the frequency bandwidth of their song and bats to lower the frequency of echolocation calls (Snell-Rood 2012), suggesting that climate change has the potential for direct effects on the evolution of these signals.

In addition to changes to the environmental acoustic transmission channel, in some organisms the animal’s acoustic signal production changes with temperature: warmer temperatures increase insect metabolism, and crickets, for example, increase their calling rate when temperatures rise (Walker 1962).

#### Other airborne sensory channels

Chemical transmission in air is also affected by temperature, wind, and humidity. Higher temperatures can increase the volatility of chemicals, decreasing the duration of pheromonal signal transmission. Higher temperatures make ant trail pheromones less stable (van Oudenhove et al. 2011), making it harder for the ants to follow their trails. This may also cause changes to community relationships, since dominant Mediterranean ant species tend to rely on pheromones more than do subordinate species, making them more susceptible to warming temperatures (van Oudenhove et al. 2011). Wind and humidity also can affect the speed, direction, and distance of chemical dispersion.

Indirect effects of climate change can also affect terrestrial animal communication. Increased fire, drought, floods, and other manifestations of the current and predicted extreme weather can be expected to each affect communication channels differently, some causing increased acoustic noise and others increasing chemical or visual noise with consequent compensation required by individuals attempting to communicate in that environment. As discussed above, the ability to shift between sensory channels when noise is encountered in one channel will play an increasingly important role in the success of organisms under new climate regimes.

### Summary: climate change as a potential driver of signal evolution

In the section above, I presented climate change as an example of the type of rapid environmental change that has the potential to drive the evolution of signaling behavior, and by extension, sexual selection and even speciation. I reviewed avenues by which climate change can affect communication channels, with the potential to impact the evolution and use of multimodal communication. I urge researchers to tackle these issues. The reality of deep and profound environmental effects from climate change is now clear (Climate Central 2012). Studying how organisms respond to these changes will allow us to better predict future biodiversity and understand how behavior evolves. Although in many cases, we may not have data from before the climate began its current phase of rapid change, we should still begin longitudinal studies now, expecting that we are near the beginning of a steep curve of upcoming change. In this way, we can collect data that reflects the early stages (relatively “pre-change”), for comparison with data from later stages. Observational and experimental data on, for example, the usage of different signal modalities in different environments under different conditions, should be collected on a regular basis to learn how organisms respond to environmental changes and whether they can adjust communication systems and strategies quickly enough to survive.

## Conclusion

The field of multimodal communication is growing quickly and becoming an important contributor to our understanding of the evolution of behavior. There are many opportunities to develop the field in valuable directions. In this essay, I have discussed a number of areas of multimodal communication research that may be fruitful to pursue, focusing in particular on the topic of whether multimodal signaling specially prepares organisms to survive rapid environmental change, due to the ability to switch between channels when one channel becomes too noisy (termed a “multimodal shift”). As examples of environmental change, I addressed urbanization and climate change and how these may impact animal communication; I urge researchers to study this. More broadly speaking, the integration of behavioral work with new techniques in molecular ecology and evolutionary genomics will allow us to address longstanding questions about the proximate basis of evolution and behavior (Ellegren 2008). In addition to integrating behavioral work with mechanistic approaches, it will continue to be important to integrate behavior with theoretical and mathematical modeling. Technical improvements are also important to pursue in behavioral work: we should continue to develop new tools for studying multimodal communication, such as computer-animated images (Woo and Rieucau 2011) and video playbacks for laboratory presentations of visual and auditory information (including vibration; see Uetz and Roberts (2002)), as well as three-dimensional robotic animals for field playbacks of visual and auditory information (e.g., Narins et al. 2003; see reviews in Partan (2004b) and Patricelli (2010)). In addition, given the importance of olfactory information in animal communication, advancing methodologies for studying olfaction in concert with the other signal channels will be helpful. Additional suggestions for future work are discussed by Partan and Marler (2005), Bro-Jørgensen (2010), Hebets (2011), van der Sluijs et al. (2011), and Munoz and Blumstein (2012).
